# Evaluating Large-Scale Integrated Care Projects: The Development of a Protocol for a Mixed Methods Realist Evaluation Study in Belgium

**DOI:** 10.5334/ijic.5435

**Published:** 2020-09-24

**Authors:** Geert Goderis, Elien Colman, Lucia Alvarez Irusta, Ann Van Hecke, Benoit Pétré, Dirk Devroey, Elias Van Deun, Kristof Faes, Nathan Charlier, Nick Verhaeghe, Roy Remmen, Sibyl Anthierens, Walter Sermeus, Jean Macq

**Affiliations:** 1 Academic Center of General Practice, Department of Public Health and Primary Care, KU Leuven, Kapucijnenvoer Leuven, BE; 2 Department of Primary and Interdisciplinary Care (ELIZA)—Centre for General Practice, Faculty of Medicine and Health Sciences, University of Antwerp, Doornstraat Antwerp, BE; 3 Department of Public Health and Primary Care, University Centre for Nursing and Midwifery, Ghent University, Ghent, BE; 4 Department of Nursing, Ghent University Hospital, Ghent, BE; 5 Institute of Health and Society (IRSS), Université Catholique de Louvain, Clos chapelle aux champs Brussels, BE; 6 Public Health Department, University of Liege, Quartier Hôpital, Avenue Hippocrate, Liège, BE; 7 Department of Family Medicine and Chronic Care, Vrije Universiteit Brussel, BE; 8 Leuven Institute for Healthcare Policy, KU Leuven, BE; 9 Research Group Social and Economic Policy and Social Inclusion, KU Leuven, Parkstraat, Leuven, BE

**Keywords:** realist evaluation, mixed methods, integrated care, chronic conditions, health systems research

## Abstract

**Background:**

The twelve Integrated Care Program pilot projects (ICPs) created by the government plan *‘Integrated Care for Better Health’* aim to achieve four outcome types (the Quadruple Aim) for people with chronic diseases in Belgium: improved population health, improved patient and provider experiences and improved cost efficiency. The aim of this article is to present the development of a mixed methods realist evaluation of this large-scale, whole system change programme.

**Methods:**

A scientific team was commissioned to co-design and implement an evaluation protocol in close collaboration with the government, the ICPs and several other involved stakeholders.

**Results:**

A protocol for a mixed methods realist evaluation was developed to gain insights into the mechanisms that foster successful results in ICPs. The qualitative evaluation proposed will be based on the document analysis of yearly ICP progress reports, selected case studies and focus group interviews with stakeholders. Processes and outcomes of all the projects will be monitored using indicators based on administrative data on population health and the quality and costs of care. A yearly survey will be organized to collect data on patient-reported outcomes and experiences and on provider-reported measures of inter-professional collaboration and proper wellbeing. Using both quantitative and qualitative data, we will develop theories about the mechanisms and the associated contextual factors that lead to integrated care and the Quadruple Aim outcomes.

**Discussion:**

The objective of this study is to deliver policy recommendations on strategies and best practices to improve care integration in Belgium and to implement a sustainable monitoring system that serves both policy makers and the stakeholders within the ICPs. Some challenges due to the large scale of the project and the multiple stakeholders involved may impede the successful implementation of this proposal.

## Background

Chronic conditions can be defined as permanent disorders, usually that have a slow progression and that are ‘*expected to require a long period of supervision, observation or care*’ [1]. Chronic diseases are the leading cause of mortality and are associated with 68% of all deaths worldwide [2]. Moreover, nearly 40% of people aged 65 and older present with multi-morbidity, i.e., are affected by 2 or more chronic conditions [3]. The ageing, often multi-morbid population and the associated rising costs challenge current health care systems and may even be a threat to their sustainability [4,5]. The current systems remain ill-suited to meet the care needs of people living with chronic conditions due to the fragmentation of services, inefficient use of financial resources and lack of coordination between care providers [6,7]. To address this challenge, the integration of health services has been promoted in many countries. However, most studies deal with integrated care interventions in a local setting and include a rather limited number of people [8,9,10,11,12]. It is well known that such innovations and programmes often have difficulties scaling up to provide sustainable, long-term interventions on a societal, whole-system level [13,14,15]. Large-scale, whole-system change programmes encounter unique dynamics and difficulties. They interact with the prevailing structures, schemes, processes and behaviours of the existing systems, which are systems that should be considered complex adaptive systems [16,17]. Therefore, these policy programmes are in need of a proper evaluation with a solid and scientific design [18,19,20].

The Belgian federated government launched such a programme plan in February 2016, the Belgian plan of *“Integrated Care for Better Health”* [21]. Its ultimate goal was to improve the Quadruple Aim objectives for people with chronic diseases, namely, improved outcomes of population health, improved patient and provider experiences and better cost efficiency [22,23,24]. Therefore, it designated 12 large-scale Integrated Care Program pilot projects (ICPs) scattered all around the country (see **Box 1**). The aim of this proposal is to engage in an in-depth evaluation of both the government plan itself and the achievements of the 12 ICPs. General recommendations exist on how to assess integrated care, but the “*application of these recommendations for a comprehensive assessment of the deployment of IC services in real-life scenarios is clearly an unmet need*” [25]. Conventional evaluation methods such as randomized control trials are ill-adapted to study the uncertain, unpredictable, and dynamic changes in complex systems [26,27]. A mixed methods study seems to be the appropriate approach for this evaluation; whereas a quantitative evaluation allows for the determination of whether any relevant change has been induced, an in-depth realist evaluation delves deeper to examine what works, for whom, in which circumstances and why [28]. Such realist evaluations draw on theories, allow us to build explanatory models and make use of quantitative and qualitative data in a mixed methods design [25,29,30].

**Box 1: The Belgian plan of “*Integrated Care for Better Health*”.**

The Belgian plan is based on the model of integrated care by the World Health Organization [31]. Twelve ICPs were designated in this plan. These ICPs are instruments to create community-based networks [32]. ICPs are both large-scale implementation projects and test cases for future scaling-up to the entire country. Each ICP covers a geographical region between 75,000 and 360,000 inhabitants, and includes approximately 10% chronically ill people [33]. ICPs are required to work within the framework of the national plan [34], including complying with the obligation to implement 14 predefined components of people-centred integrated care. The 14 components involve changes at the micro and meso levels [35].

The 14 components are as follows:

✓ patient empowerment,
✓ support for informal carers,
✓ case management,
✓ socio-professional and socio-educational reintegration,
✓ a focus on prevention,
✓ multidisciplinary consultation and coordination,
✓ extra-, intra- and transmural care continuity,
✓ valorisation of the experience of patient associations, family associations and health insurance funds,
✓ integrated patient records,
✓ the use and dissemination of multidisciplinary guidelines and protocols,
✓ the development of a quality culture,
✓ the adaptation of financing systems,
✓ the stratification of risks in the population and mapping of the environment,
✓ change management guided by the ICP governance structure

Together, the 14 components represent 3 dimensions of the integrated approach:

– Integration of the patient and his or her environment.
– Integrated multidisciplinary coordination, consultation and information sharing.
– A different approach to health and social care, e.g., the development of a quality culture.

Within this framework, ICPs have some freedom, e.g., to make decisions regarding their specific goals, their target population and their actions. This freedom of choice has led to an important heterogeneity among ICPs, especially with regard to their target populations and action plans. (See addendum).

However, literature on realist evaluations of large-scale change programmes is scarce. The nature, scale and long-term perspective of such projects may impede or hamper the possibility of obtaining solid and valid research results. This paper addresses the question of how to develop a realist evaluation of a large-scale, nationwide policy plan that addresses a whole-system transformation towards the increased integration of health care services.

## Methods: Co-creation of the evaluation framework

A scientific team, FAITH.be (Federated consortium for the Appraisal of Integrated care Teams in Health in Belgium), was commissioned to (1) design and implement a framework to evaluate the ICPs and (2) support the ICPs in self-evaluation. The general objective of the scientific team is to identify best practices and to inform policy makers regarding how to evolve towards integrated care in Belgium. Faith.be consists of research teams from six different universities, including researchers specializing in public health, general practice, nursery and social sciences.

Faith.be is not entirely autonomous. It is committed to work with the commissioners funding the design, implementation and evaluation protocol of the projects. As the evaluation framework should suit the goals of different stakeholders, its design was constructed through a co-creative process involving academics, governmental agencies and ICPs. Co-creation can be defined as the “*collaborative generation of knowledge created by academics working alongside stakeholders from other sectors*” [36]. Co-creation is considered a necessity to the development of research projects with high societal relevance and impact [36,37]. Regarding the quantitative outcome analysis, it was agreed that the evaluation of the ICPs would include a common base that included indicators that were common for all ICPs. Above that common base, a set of outcome and process indicators would be constructed for each specific ICP. Reporting these specific sets of indicators is beyond the scope of this article. The large-scale setting excludes the possibility of prospectively collecting data for all the included patients. Therefore, the quantitative evaluation will almost exclusively be based on routinely collected data, mostly insurance data. These data, managed by the Inter Mutualistic Agency (IMA), will be made accessible and merged with other data, (e.g., Patient Reported Outcome Measures [PROMs] and Patient Reported Experience Measures [PREMs]) by a trusted third party, Healthdata.be. Healthdata.be is part of the scientific government agency Sciensano. It offers a platform for pseudonymized patient data to facilitate the data exchange between researchers and healthcare professionals. It incorporates both technical and non-technical measures to ensure the protection of the patient’s privacy, as well as that of the caregiver, and assures medical confidentiality. Since the data are pseudonymized, merging them at the patient level while maintaining the privacy of the patients is possible. Researchers must request the permission of the Belgian Data Protection Authority and complete a detailed form indicating the precise data that are needed and the purpose for which they will be used. Moreover, only selected dedicated researchers receive a key to access the data warehouse that contains the merged data. They can never download a database. Small cell analyses are performed, and access to certain records is prohibited if necessary to guarantee the privacy of the patients.

We acknowledge that the study protocol may need further adaptation over time because it was impossible to ensure the practical feasibility of all the planned actions.

Indeed, a defining feature of co-creation is its emergent and adaptive nature, which makes detailed pre-specifications of interventions and outcome measures impossible [26]. During the design process, some parts of the protocol, e.g., the ROMs and the PREMs surveys, already underwent important changes. It appears that Faith.be is not allowed to contact patients because of privacy matters. Therefore, to reach the patients, the health insurance funds and the dedicated providers of the patients will be asked to contact the patients to complete the questionnaire. It must be noted that each change in the research protocol must be accompanied by a revision of the privacy agreement. Another example is the annual report with which ICPs have to document their proceedings. The questions listed in this annual report have been adapted multiple times, in collaboration with the authorities and the ICPs.

In summary, Faith.be is dependent on multiple organizations and stakeholders for the implementation of the protocol. Therefore, regular meetings and weekly email discussions have been organized between Faith.be and the involved stakeholders. Additionally, the government set up an agency, the Inter-Administrative Cell (IAC), to accompany and supervise both Faith.be and the ICPs. All decisions regarding the design and implementation of the evaluation protocol take place in direct collaboration with the IAC. In the following sections of this paper, an overview is given of the content of the research protocol, as agreed upon by the various stakeholders.

## Results: the research protocol

### Research questions

Based on the requirements of the government plan, Faith.be defined common research questions for each ICP, all related to the Quadruple Aim:

1. What changes have occurred over time in the health of the population of the projects?
2. What changes have occurred over time in the cost of care, more specifically regarding the indicators of low- and high-value care?
3. What changes have occurred over time in the Patient Reported Experience Measures (PREMs)?
4. What changes have occurred over time in the indicators of professional functioning and wellbeing?

Furthermore, an in-depth explanatory analysis will answer questions regarding the implementation of these complex interventions in their particular loco-regional contexts and the association between the implementation processes and the achievement, or lack thereof, of the predefined outcome objectives. These questions are as follows:

5. What factors enabled an ICP to achieve its Quadruple Aim objectives and the 14 components of integrated care or hindered it from doing so in its local context? Which objectives were achieved? Which objectives were not achieved? For whom? How and why? To which unintended consequences has the ICP led?
6. Which adaptations were needed to reach those objectives? Why and when were these adaptions made and with what means/resources?
7. How do healthcare professionals in ICPs experience the ICP? More specifically, based on Normalization Process Theory,
  - Do they understand and agree with the programme (*Coherence*)?
  - Are they led and recognized when becoming involved with the programme (*cognitive participation*)?
  - Do they see positive changes in their work activities (*collective action*)?
  - Do they have the means to learn from what they are doing (*reflexive monitoring*)?

### Implementation analysis

#### A framework for building an explanatory theory

The implementation analysis aims to answer research questions 5, 6 and 7 using both qualitative and quantitative data. The analysis of the qualitative data allows for a deeper examination of how success was achieved or how failure occurred. The objective of the analysis of quantitative data is to determine whether success was achieved. First, guided by the RAMESES II framework [38,39,40] and starting from Normalization Process Theory (NPT) [41,42,43], an initial programme theory explaining the logic of the implementation of the national plan towards integrated care will be constructed. In summary, the NPT characterizes the social mechanisms implicated in implementation processes and explains their operation. Hence, it explains why an ICP does or does not reach its objectives regarding the improvement of care integration and the Quadruple Aim objectives in its local and particular context. In doing so, it provides a good starting point to explain how changes in the context of an ICP may influence the coherence, cognitive participation, action and reflection of the actors involved at its micro, meso and macro levels. This theory will be tested and refined by a multiple case study and an analysis of all the ICPs.

#### A multiple, embedded case study of 3 loco-regional programmes: data collection

To identify explanatory theories about why a programme is successful in its given context [44], a multiple, embedded case study approach will be used. Three case studies were selected based on their region, level of urbanization, target population, action plan content, governance structure, and the balance between primary and secondary care. The aim of these case studies will be to provide in-depth insights into the implementation of the ICPs. Data for these cases will be gathered through document analysis, significant change reporting, focus groups with stakeholders involved in governance, care providers and patients. If needed, additional individual interviews will be performed.

##### Document analysis

All the relevant documents (e.g., action plans, Gantt charts, contracts, and annual reports) concerning the three case studies will be systematically selected, analysed, coded and synthesized. In the first stage, the document analysis will particularly focus on the identification of the programme theory: how actions and activities should lead to better integration of care and better Quadruple Aim outcomes according to the ICP action plan. Once the programme theory has been described, the document analysis will focus on the development and adaptation of the projects. The annual reports of the ICPs will be key documents in this analysis; each year, the ICPs have to complete an open-ended questionnaire on governance, process evaluation, their implementation of the action, components of integrated care and programme changes. This questionnaire will be developed in co-creation with the authorities to suit both the purpose of administrative follow-up and scientific evaluation.

##### Yearly focus groups

To obtain more in-depth information, yearly focus groups of 10–12 participants, including the coordinator(s) and main stakeholders involved in the project’s governance, will be organized. The focus groups will aim to determine the perceptions of the stakeholders about the level of the implementation of the programme and will employ an interview guide based on the *NOMAD tool.* This tool was made by the developers of Normalization Process Theory and has proven useful for these purposes [45,46]. Additional questions will be added based on the document analysis and the content of the annual reports.

##### Significant changes

It is requested that ICPs report significant events (e.g., changes that might have a significant impact on the governance of the projects), and these reports will be evaluated using the “Most Significant Change Technique” [47,48]. This technique aims to collect descriptions of significant changes (significant change stories) and select the most important of these, analysing the most significant changes with the most important stakeholders to draw general insights [48].

If more information is needed to identify the programme theory, implementation proceedings or significant events, additional individual interviews with coordinators and other people involved in ICP governance will be performed.

#### Data collection with each ICP

Qualitative data from each ICP will be collected. First, each project that was not selected for the multiple embedded case study will be asked to complete the annual report. Moreover, members of the scientific team will organize learning community meetings. The goal of these meetings will be to connect all the ICPs, the supporting government body and the scientific team to share knowledge, identify best practices and learn from each other. All the project coordinators and their main stakeholders will be invited to freely exchange information about their difficulties and the knowledge that they have gained, as well as to share good practices. The desired outcome of this learning community is support of the projects, but the content of these meetings will also be very valuable for the analysis of the implementation. Together, these various methods of qualitative data collection should allow us to collect in-depth information about the context, mechanisms and aspired outcomes of all ICPs.

The discussions of the stakeholder focus groups selected in the case studies as well as the content of the learning community meetings and the interviews with the stakeholders will be recorded and transcribed with the consent of the participants. For each ICP, a responsible researcher will be appointed who will continuously add information to the analysis as it is gathered from the document analyses, focus groups and interviews. All the data sources will be thematically coded based on NPT, and QSR NVivo12 for teams will be used to support the data management. For each ICP, a responsible researcher will be appointed who will continuously add and summarizes the information as it is gathered during the implementation of the document analyses, focus groups, interviews and significant events in a summary ICP record. These ICP records will allow us to keep an audit trail of the data collected and of the standardization of the data collection and analysis. This will be used as a coded database of qualitative data, in which all relevant research findings will be displayed, along with the precise (dated) sources from which the data came.

### Quantitative analysis

#### Quadruple Aim monitoring system

The final aim of the quantitative framework will be to set up a sustainable Quadruple Aim evaluation monitoring system. This monitoring system will be used to evaluate the ICPs on a regular basis and to enhance evidence-based decision-making within the ICPs. This monitoring system will rely as much as possible on the routinely collected data available in different administrative data sources. In Belgium, reimbursed healthcare consumption and hospital data have been routinely collected for many years, and the number of data sources available continues to increase. Healthdata.be will link these various databases at the patient level using the encrypted national identification number.

#### Setting, participants and data collection

##### Populations of interest

The setting of the study is defined as the 12 ICPs. The Belgian population outside the areas of these ICPs will be used as a pool for the comparison population. As shown in Table 1, two different types of samples will be defined. First, as the ICPs cannot be considered as an intervention directed only towards specifically included people, we hypothesize that the ICPs will have an impact *on the whole population.* As such, a ‘target population’ (Population A) will be defined for each ICP. This ‘target population’ will consist of the people for whom the services offered by the ICP are intended as defined in the action plan. However, not all individuals in the target group will eventually be included in the ICP. Therefore, the part of the target population that has actually been included in the ICP and those who have actually been offered ICP-related services will be defined as the ‘included population’ (Population B).

**Table 1:** Overview of data sources available for each patient population under study.

Study Population		Data on sickness funds (IMA)	GP data	Hospital data	BELRAI	PROMs & PREMs	Statistical analysis

Population A: Target population	The population for whom the developing services that will be offered by the ICP are intended and who are living in the specified geographical region	X (aggregate level)					Cross-sectional time series & longitudinal cohort study
Population B: Included patients	The part of the target population that has actually been included in the ICP and who have been offered ICP-related services	X	X	X	X		Longitudinal cohort study

Baseline data (T0) will be collected from the period before the introduction of the ICPs, i.e., 2016. In 2016, the first call for projects was launched, but the definite approval of the 12 ICPs only took place in 2017, and the projects did not start to accept patients until the autumn of 2019. The only available data source for the T0 period is the IMA database. Since the aim of this endeavour is to set up a permanent monitoring system, no exact endpoint (T1) has been defined. The intention is to organize annual data collections in the long run.

##### Data sources differ according to the population

Table 1 also gives an overview of the available data sources for each aspect of the Quadruple Aim and for the different samples. The primary data source for the outcome evaluation will be the Inter Mutualistic Agency (IMA-AIM) data warehouse, which contains data on all reimbursed health interventions, e.g., general practitioner and specialist visits, technical and diagnostic interventions, hospital and emergency admissions, and medication. These data are available for the whole target population (population A, see Table 1). For the included population (population B), the long-term aim is to link individual IMA data with other data sources, such as hospital data, data extracted from General Practitioner Electronic Medical Files and survey data from the Belgian Resident Assessment Instrument (BELRAI) screener [49,50]. It should be noted that BELRAI results will enable a risk and frailty classification [51]. To complement the administrative data, both patients and care providers involved in the ICPs will be surveyed. An invitation letter will be sent or given to all included patients. This letter will include a link to an encrypted, secured website with the PROM and PREM questionnaires, namely, the 5Q-5D-5L questionnaire [52] and the Patient Assessment of Chronic Illness Care (PACIC) questionnaire [53]. Both questionnaires have been translated and validated in French and Dutch.

A subsample of professional care providers (Population C) involved in an ICP will be asked to participate in a web survey to evaluate (a) job satisfaction, which will be assessed by a portion of the RN4Cast-research questionnaire [54]; (b) burnout, which will be assessed by the UBOS questionnaire [55]; and (c) relational coordination [56]. Due to the small sample size and/or the expected high turnover of patients and professionals, it will not be possible to perform a longitudinal study using this data.

#### Indicators

##### Quadruple Aim indicators

A set of Quadruple Aim indicators was developed. In total, nearly 300 parameters were selected for analysis in consultation with all the stakeholders and based on the consensus in the literature on the Quadruple Aim [57,58], action plans of the ICPs, government objectives, existing methodology and criteria on quality indicator selection [59,60] and feasibility. Table 2 gives a non-exhaustive selection of the most relevant indicators.

**Table 2:** Overview of the most relevant Quadruple Aim indicators and samples of interest.

Domain and indicator	Numerator	Denominator	Data Source	Sample

**Population Health**				
Mortality	Number of deaths	Total population & population stratified by age and subgroups	IMA	A/B
Occupational disability	Number of people who have reported a lapse in professional occupation due to health issues during the last 12 months	Active population stratified by the criteria mentioned later	IMA	A/B
Activities of daily living (ADL) & instrumental activities of daily living (IADL)	Number of people with ADL > 3/IADL after 1 year	Total number of patients stratified by subgroups	BELRAI	B
Mobility, self-care, usual activities, pain & discomfort, anxiety & depression	Number of people with no or slight problems	Total number of patients in Sample C	EQ-5D-5L**	B
Quality of life on a visual analogue scale	Number of people with a self-reported quality of life > 80	Total number of patients in Sample C	EQ-5D-5L	B
**Patient Experiences**				
PACIC domains (4): Patient activation; delivery system design; goal setting; problem solving	The average percentage of patients who respond “most of the time” for each domain	Total number of people in the sample	PACIC**	B
**Cost efficiency, care with high and low value and equity**				
ED visits and hospitalization through the emergency department (per 1000) during the last 12 months	Number of episodes during the last 12 months	Total population & population stratified by subgroups (Rate)	IMA	A/B
Number of early 30-d readmissions (per 1000) in the last 12 months due to Xi disease	Number of early 30-d readmissions in the last 12 months	Total population & population stratified by subgroups (Rate)	IMA	A/B
Number and indices of n° of specialized visits/GP visits***	Number of outpatient specialized visits in the last 12 months in a given person’s profile	Total population & population stratified by subgroups & by SES**** (Rate)	IMA	A/B
Number of dental visits per year****	Number of early 30-d readmissions in the last 12 months	Stratified by SES		
Proportion of patients using ≥5/10 drugs (per 1000), last 12 months, due to any cause	Number of patients using ≥5/10 drugs, last 12 months, due to any cause	Total population & population stratified by subgroups	IMA	A/B
Proportion of patients with ≥2 ER consultations during a 6 months period, last 12 months	Number of patients with ≥2 EM consultations during a 6 months period, last 12 months	Total population & population stratified by subgroups	IMA	A/B
**Professional Well Being**				
Indicator	Numerator	Denominator		
Relational coordination	Number of providers who indicate that they often or always collaborate	Total number of providers	RC survey	C
Job satisfaction	Average number of providers who respond “Satisfied” or “Very satisfied” for select indicators of job satisfaction	Total number of providers	4N4Cast	C
Burnout	Average number of providers who respond “Often”, “Very often” or “Always” for select indicators of UBOS	Total number of providers	UBOS	C

* Population A includes population B. ** Self-Reported. **** Serving as an indicator for equity [62]. **** SES = Socio Economic Status.

The evaluation of population health will be based on diagnoses and specific care needs. Costs will be calculated using specific claims codes (called medical nomenclature codes) and will be interpreted and aggregated into specific cost components with the intention of monitoring potential transfers of a patient’s healthcare cost to different healthcare professionals or healthcare providers (e.g., a transfer from hospitalization cost to in-home care cost). Efficiency and equity will be evaluated indirectly according to the value-based healthcare concept (VBHC). The use of “high-value care” (in contrast to “do-not-do care” or “low-value care”) will serve as a proxy measure for efficiency and equity. Low-value services are related to misuse, overuse or underuse of health services. Do-not-do care refers to the NICE ‘do not do’ recommendations [61]. High-value care that is not used by people in need with given characteristics (a specific disease or a specific socioeconomic status) will be used as a proxy for equity. In addition, a care trajectory analysis for subgroups of patients will be performed. This means that based on the literature and expert opinion, ‘high-value’ sequences of care will be defined according to their expected impact on (better) health outcomes and/or (lower) costs. An example of high-value care is a general practitioner visit within 7 days after hospitalization for people with frailty. Once defined, these sequences can be analysed and compared over time and between ICPs.

##### Process Indicators

Process indicators will allow us to evaluate the reach of the ICPs and determine which specific actions that individual patients are benefitting from within the ICPs, which are important elements of the implementation analysis. Some of these indicators will be calculated based on external data sources (see Table 3), and other indicators will be calculated based on the data collected by the ICP. Some of the process indicators listed in Table 3 will be collected among all ICPs. For the activities that are not common to all ICPs, project-specific indicators will be collected.

**Table 3:** Key process indicators common to all ICPs.

Action	Indicator

Inclusion	% of included patients (with informed consent)
Medication review	Relative share of medication that was prescribed using the International Non-proprietary Name (INN)
Training sessions for professionals	Number of training sessions organized
	Number of professionals (per type) who participated
Training sessions for patients	Number of training sessions organized
	Number of patients who participated
Case management	Number of patients in a case management trajectory
Case management	Case load: Number of patients in case management/number FTE case managers
Care pathways	Number of pathways
	Number of patients in each pathway

##### Difference-in-differences analyses

The question of *whether* significant change in relevant Quadruple Aim indicators has occurred will be evaluated through a before/after comparison and a difference-in-differences (DID) analysis of the target population and the included population of the ICPs with a non-ICP comparison group. This will only be possible for data from the IMA warehouse (see Tables 1 and 2) since those data are available for the whole Belgian population. As such, all people living in Belgium outside an ICP region constitute the pool for comparison. Similar quasi-experimental designs have been used during the evaluation procedures of other natural experiments [63], defined as deliberate events, programmes or (complex) interventions. *“Exposure to the events or interventions has not been manipulated by the researcher”* in these designs [63]. Direct matching, propensity score matching, or group comparison with adjustment for confounders are possible comparison methods. The aim is to reduce the observed and unobserved confounding factors. Since this model deals with dynamic cohorts (new participants will be added to the target, the included and the comparison populations over time) for which it is not clear how to implement matching techniques [64,65,66,67,68], we opted for a group comparison by means of a generalized linear model for correlated longitudinal data and adjustment for confounders.

### Integrative mixed methods: context-mechanisms-outcomes analysis to explain the observed changes

To obtain insight into *how* Quadruple Aim outcomes will be reached, it will be necessary to combine qualitative and quantitative analyses. Mixed methods bring together qualitative and quantitative approaches into a single study and rely upon the complementary strengths of each approach to address the study questions [69]. Quadruple Aim outcomes must be considered ‘distal outcomes’ [70] and are not expected to change significantly in the first years after the start of the implementation [71]. In the meantime, the process indicators can be collected and analysed to explore the short-term changes. For this, we will use realist evaluation to identify context-mechanisms-outcomes configurations (CMOCs) explaining whether and to what extent the interventions of the programmes, or parts of these programs, were successful. Through the analysis of both the qualitative and quantitative data collected from the patients, care providers and coordinators of the ICPs, mid-range theories will be developed and tested. The aim of these theories will be to explain the contextual factors and mechanisms by which ICPs may evolve towards better care integration and improved Quadruple Aim outcomes [72]. Qualitative analyses will be based on the results of focus groups, significant event reports, interviews, learning community meetings, key documents such as the annual reports and other documentation of the ICPs. The insights, as they develop, will constantly be compared among all ICPs to allow for the identification and testing of the logic of the interventions in each context [73]. For example, explorative CMO multivariate analyses will be performed comparing the evolution of different ICPs, and these results will be discussed during reflective meetings within the learning communities. This will lead to the further refinement of our theory and the identification of context-mechanisms-outcomes configurations explaining the success– or failure of the programme interventions. These steps will be refined through their comparison with the results of other ICPs; repeated discussions within the multidisciplinary research team; and consultation with international reviewers, stakeholders of the ICPs and the government. The order of these steps will, however, vary, as realist evaluations intertwine between theoretical concepts emerging from qualitative analyses and literature with preliminary findings from empirical data. The analysis of the data will lead to insights that will be fed back to the ICPs and can help them in their work.

As such, the quantitative outcome analysis and the realist evaluation are combined in a sequential triangulation with a cyclical nature [74].

## Discussion

This paper describes an evaluation protocol for large-scale policy initiatives that are intended to induce a sustainable shift in the health care system towards increasingly person-centred integrated care. Since these initiatives interact with the current structures, processes and behaviours in complex adaptive systems, a mixed methods realist evaluation seems to be a necessity in the evaluation of these macroscale change programmes. The presented approach entails a combination of both qualitative and quantitative data as well as process and outcome indicators and involves key stakeholders for the interpretation of results.

Quantitative data allow for the evaluation of whether a significant change has occurred in relevant Quadruple Aim Indicators. Due to its scale and long-term timeline, this evaluation will be almost entirely based on routinely collected data. Once set up, this monitoring system can later also be moved to other regions. This approach will reduce the burden on health professionals and ensure the long-term sustainability of the evaluation system. The combination of quantitative and qualitative analyses in a mixed method design will allow an explanatory model to be built explaining which actions were successful in the ICPs, for whom, under what circumstances and why. This approach is expected to deliver useful insights into the interaction between context and the mechanisms within a complex adaptive system that will eventually lead to integrated care and improved Quadruple Aim outcomes. This is pivotal for identifying effective, context-related strategies and for understanding how these strategies can be applied or adapted to other contexts [75,76,77].

A large number of integrated care projects address small- and middle-scale interventions and sample sizes ranging from 10 to approximately 2,000. Study designs include before/after trials, randomized controlled trials, quasi-experimental trials, mixed methods and qualitative studies [8,10,78,79]. The studied indicators include health outcomes and care utilization [78,80,81], process indicators [82,83], costs (economic evaluations) [84,85] and CMO mechanisms (realist evaluations) [86,87,88,89]. Literature on well-designed evaluations of large-scale population-based programmes is scarce. Best et al. found 84 empirical studies of large system transformation [90]. However, many of these studies did not apply a well-developed quantitative design, e.g., lacked a comparison group [91,92]. Only some large-scale policy programmes, such as Gesundes Kinzigtall and the North-West London Integrated Care pilot project, have been cited in the literature for their well-developed evaluation design [91]. While the evaluation of Gesundes Kinzigtall [93] was mainly quantitative with a quasi-experimental design, North-West London used mixed methods to evaluate the project [94]. In particular, the latter project is interesting for the Faith.be design because it also integrated service utilization and costs based on qualitative data [94,95]. However, unlike the North-West London project, the Belgian plan has developed 12 different and heterogeneous projects. This heterogeneity, together with the large scale of the project, the multiple stakeholders and the interdependence of everyone included in the project, involves certain challenges. Co-creation, shared decision making and collaboration with multiple partners are indeed necessary to develop research with societal relevance and to “get things done” when implementing the protocol. However, in practice, the co-design and implementation of a protocol is a difficult, intensive and time-consuming process. The research consortium is dependent on federal, regional and local stakeholders for data availability, accessibility and quality. Continuing disagreements and delays during decision making and implementation are serious risks. These delays are correlated with the complexity of the programme being designed and its evaluation, the number of involved stakeholders and the degree of interdependency between those stakeholders. An evaluation of a complex project such as Integreo is already complicated. Each additional layer of complexity, e.g., the decision to merge data on a patient level, may hamper or block the implementation of the programme. Therefore, the design of the evaluation should be kept as small and simple as possible. Additionally, it should be planned in different phases, starting with a more ‘basic’ evaluation in the beginning and followed by a more thorough analysis later. Additionally, the governance and consultative structures of such projects should be kept as simple as possible, carefully considering the trade-off between technical and political necessity on the one hand and efficient decision making on the other.

The “co-creative triangle” between policy makers, their agencies, researchers and ICP stakeholders also introduces challenges and limitations. First, co-creation with 12 different ICPs, each containing approximately 50 different organizations and several coordinators, is truly challenging. Although regular communication and joint meetings exist, most ICPs consider Faith.be a ‘foreign body’ and thus external to their project. Because of this, Faith.be has not truly been involved in the design of the projects, and the projects have not truly been involved in the design and practicalities of the research protocol. Most IPCs express difficulties of ownership regarding the evaluation protocol. Moreover, some projects have been developed by universities and are supported by proper academic researchers who may desire to develop their own evaluation. To foster co-creation, it would have been better if each project would have delegated one skilled person to join the Faith.be research consortium.

Second, the co-creative relationship between a research team and the commissioners must be considered carefully. Commissioners are often in a position in which they need rapid and straightforward results in the form of a summative evaluation. This is because they have to make decisions about the continuation and financing of the projects. However, the complex reality and nature of these projects often hamper the possibility of delivering rapid and straightforward results. Moreover, studies often adopt an understanding attitude aiming for a formative instead of a summative evaluation. Managing this tension between researchers and commissioners – a well-known problem referenced in the international literature [71] – is challenging. Therefore, open and transparent discussions about mutual expectations, priorities, vision and the roles of each party should occur when the project begins and regularly afterward. Each party should form expectations that keep the reality and complexity of large-scale projects in mind, as well as the possibility of drawbacks, obstacles and delays. Regular reality checks seem to be a necessity.

The qualitative part of the evaluation will rely on documents provided by the ICPs that are also used by the authorities to make decisions regarding the continuation and financing of the ICPs. This may affect the way in which they present their plans and proceedings. Moreover, the supportive role Faith.be was given as a secondary task may create partiality towards the projects and thus bias the evaluation results of these programmes.

Regarding the quantitative analysis, routinely available ‘real-world data’ show a certain degree of uncertainty and a risk of bias [96]. Interpretation of the results of this analysis should be performed cautiously. However, repeated data collections over a period of several years may provide scientifically sound results on the time trends of key indicators. Other types of bias, especially selection bias, may also occur since participation in an ICP is voluntary. In addition, members of the target population living in an ICP region but not directly included in the programme may still benefit from actions taken by the project, particularly those implemented at the meso-level. Moreover, the inclusion of patients in ICPs may be based on clinical decisions (i.e., the decision of a general practitioner to include a patient in an ICP). Information bias can interact with this kind of selection bias. If unobserved confounders were part of this clinical decision, then the results may be subject to confounding by clinical indication [97,98].

## Conclusion

Developing a mixed-methods protocol to evaluate a political, nationwide change programme with the aim of increased integration of care is innovative and challenging. This realist evaluation combines routinely collected population data with process data and qualitative research. Such an approach seems necessary to evaluate where, whether and to what extent the programme has succeeded and to more deeply examine the mechanisms and contexts associated with its failure or success. However, there are serious challenges to implementing this protocol due to the large scale and heterogeneity of the projects and to the involvement and interdependence of multiple stakeholders. Further implementation of the protocol will reveal how to address these challenges and what lessons can be learned. Successful implementation should lead to valid, scientifically sound policy recommendations and enable a sustainable monitoring system.

## Abbreviations

BELRAI: Belgian Resident Assessment Instrument

CMOCs: Context-Mechanisms-Outcomes Configurations

DID: difference-in-differences

EMF: Electronic Medical Files

FAITH.be: Federated consortium for Appraisal of Integrated Care Teams in Health in Belgium

FUPS: Flawed, uncertain, proximate and sparse data

IC: Integrated Care

ICPs: Integrated Care Program Pilot projects

IMA-AIM: Inter-Mutualistic Agency

Integreo: Integrated Care for better health

NPT: Normalization Process Theory

PACIC: Patient Assessment of Care for Chronic Conditions

PROMs: Patient Reported Outcome Measures

PREMs: Patient Reported Experience Measures

RC: Relational Coordination

VBHC: Value-Based Health Care

## Author Information

Elien Colman and Geert Goderis share the first authorship and Geert Goderis is the corresponding author.
